# Attributes and Features of Context Relevant to Knowledge Translation in Health Settings: A Response to Recent Commentaries

**DOI:** 10.34172/ijhpm.2023.7908

**Published:** 2023-02-27

**Authors:** Janet E. Squires, Wilmer J. Santos, Ian D. Graham, Jamie Brehaut, Janet A. Curran, Jill J. Francis, Jeremy M. Grimshaw, Michael Hillmer, Noah Ivers, John Lavis, Susan Michie, Thomas Noseworthy, Alison M. Hutchinson

**Affiliations:** ^1^Faculty of Health Sciences, School of Nursing, University of Ottawa, Ottawa, ON, Canada.; ^2^Clinical Epidemiology Program, Ottawa Hospital Research Institute, Ottawa, ON, Canada.; ^3^Faculty of Medicine, School of Epidemiology and Public Health, University of Ottawa, Ottawa, ON, Canada.; ^4^Faculty of Health, School of Nursing, Dalhousie University, Halifax, NS, Canada.; ^5^IWK Health Centre, Halifax, NS, Canada.; ^6^School of Health Sciences, University of Melbourne, Melbourne, VIC, Australia.; ^7^Ontario Ministry of Health and LongTerm Care, Toronto, ON, Canada.; ^8^Dalla Lana School of Public Health, Institute of Health Policy, Management and Evaluation, University of Toronto, Toronto, ON, Canada.; ^9^Women’s College Research Institute, Toronto, ON, Canada.; ^10^0Department of Health Research Methods, Evidence and Impact, McMaster University, Hamilton, ON, Canada.; ^11^University College London, London, UK.; ^12^Department of Community Health Sciences, University of Calgary, Calgary, AB, Canada.; ^13^School of Nursing and Midwifery, Centre for Quality and Patient Safety Research, Institute for Health Transformation, Deakin University, Geelong, VIC, Australia.; ^14^Barwon Health, Geelong, VIC, Australia.

## Introduction

 Many authors commenting on our paper about stakeholder perceptions of context^1^ have recommended the use of complexity theory^2-6^ as a frame for understanding context as a complex system comprising many interrelated parts of a larger implementation system^7^. This larger system can be seen as composed of multiple elements (eg, context, knowledge translation [KT]) or implementation strategies (eg, facilitation), and interventions (eg, evidence-informed practices, programs, or policies) that may moderate and influence one another. The interrelationships between these elements mean that changes in one element can influence change in many other elements of the system.^7^ Harvey^4^ emphasizes the importance of developing tools that can help individuals prioritize the context attributes that should be assessed and evaluated. We agree with these commentaries. As we have posited, the first step required to understand interrelationships between context attributes or to develop the tools for prioritizing context attributes is to improve conceptual clarity of the context concept by identifying the attributes and features of context.^8^ Currently, there is inconsistency in the literature pertaining to the definition of context or its attributes.^8^

 To advance conceptual clarity of the “context” concept in relation to implementation in healthcare, we assembled a large international team of researchers and knowledge users to conduct a series of three interrelated studies (qualitative interviews with system stakeholder interviews, a concept analysis, and a secondary analysis of interviews conducted with health care professionals).^1,8,9^ The paper reporting on qualitative interviews with system stakeholders’ tacit knowledge of context^1^ is the subject of the commentaries. Using multiple methodologies, followed by a meta-synthesis of the findings from the three studies, we produced the Implementation in CONtext (ICON) framework. The resulting ICON framework conceptualizes context across three levels (micro, meso, and macro), which are further divided into six domains, 22 context attributes and over 100 example features.

## Pragmatist Paradigm and the ICON Framework

 Many of the commentaries raised the issue of how worldview influences the development and proliferation of particular forms of knowledge.^2-6,10^ Particularly, some authors stated that the formation of a list of attributes and features of context aligns with a post-positivist paradigm,^2-6^ whereas other authors acknowledged our inductive and constructivist approach in identifying context attributes and features by interviewing system stakeholders.^6,10^ Our development of the ICON framework was rooted in a pragmatist paradigm. A pragmatist worldview depicts a pluralist perspective of truth (ontology) and knowledge (epistemology), in which truth and knowledge are perceived as objective (existing independent of one’s own perception), subjective (constructed based on an individual’s experiential context) and dictated by historical and sociocultural structures.^11^ Furthermore, a pragmatist paradigm dictates the importance of multiple sources of knowledge and the use of different methodologies to answer research questions.^11^ The three interrelated studies that informed the development of ICON and a brief summary of their results are as follows:

1. A qualitative semi-structured interview with system stakeholders (change agents/KT specialists and KT researchers) in four countries: Australia, Canada, the United Kingdom, and the United States (n = 739 interviews) to elicit tacit knowledge of what they perceived as context. This study identified 66 unique features organized into 16 context attributes.^1^
2. A concept analysis (n = 70 included studies) that searched for published articles that provided a definition of context or described its attributes in biomedical and social science databases. This study identified 201 unique features of context, 89 features were reported in two or more articles. The authors organized the 89 shared features into 21 context attributes.^8^
3. An exploration of what context attributes influences healthcare professionals’ research use through a secondary analysis of qualitative semi structured interviews (n = 7145 interviews) informed by the Theoretical Domains Framework.^12^ The interviews were undertaken in 10 Canadian studies and one Australian study and examined healthcare professionals’ perceived barriers and facilitators to their research use. The study identified 62 unique features organized into 14 context attributes.^9^

Our approach aligns with Harvey’s^4^ and Pfadenhauer’s^6^ recommendations that researchers should use multiple methodologies to address conceptual problems. We agree with Pfadenhauer^6^, van Pelt and Beidas^10^ who commented that our qualitative interview study with health system stakeholders provides important insights about the context concept. In keeping with our approach to investigate both healthcare professionals’ and health system stakeholders’ perspectives on context, several commentaries^3,6,10,13^ stated that the perspective of a large group of individuals from diverse professional and social backgrounds is needed to advance conceptual understanding of context.

## How ICON Advances Conceptual Clarity of the Phenomenon of Context

 The commentaries on our study that evaluated stakeholders’ tacit knowledge of context are also relevant to the ICON framework. Gagnon^5^ questioned the need for another framework, given the number of existing frameworks that address context. However, our extensive methodology was designed to be as comprehensive as possible in identifying factors relevant to context. This enabled us to identify and include in ICON at least one attribute and 44 features that are not present in the Tailored Implementation in Chronic Diseases framework checklist^14^ (a recent and comprehensive implementation framework describing context). For example, in the studies that evaluated healthcare professionals’^8^ and health system stakeholders’ tacit knowledge,^1^ we identified the “facility characteristics” attribute (defined as the “attributes of a building or buildings designated as a site for providing healthcare”^8^ (p. 11), which is not present in the Tailored Implementation in Chronic Diseases framework checklist.^14^ Pfadenhauer^6^ in her commentary on our paper noted that despite the importance of facility and spatial context, this attribute of context is overlooked in most determinant frameworks that describe contextual determinants for implementation outcomes. We appreciate Pfadenhauer’s^6^ recognition of our detailed description of context attributes at the macro level (eg, regulatory, and legislative standards), which she stated are often under-assessed and under-developed aspects of context. Similarly we are pleased that several of the commentary authors^2-5,13,15^ appreciated our creation of a thorough list of context attributes based on our qualitative study with health system stakeholders,^1^ a list which is now even more comprehensive in ICON. We agree with Van Pelt and Beidas’^10^ assertion that a list of context attributes and their definitions can act as a shared vocabulary between researchers and knowledge users, which can further encourage collaboration amongst these groups. Harvey^4^ reported that based on the findings of the system stakeholder qualitative paper,^1^ culture and resources should be the main foci as they were consistently mentioned by system stakeholders. Her comment^4^ provides insight on how we might integrate the research evidence we compiled in developing ICON to inform decisions related to practice and policy.

## Utility of the ICON Framework

 In alignment with the pragmatist paradigm,^11^ the ICON framework can be used by researchers and knowledge users who perceive truth and investigate knowledge through different paradigmatic perspectives. Below we discuss how we envision the ICON framework can be used by those with post-positivist, social constructivist, and transformative perspectives.

###  Post-positivism Paradigm

 Post-positivism, based on the rationalist and empiricist perspective, assumes a deterministic and rational cause that is observable and measurable for every outcome.^11^ The use of an existing framework to deductively guide the formation of null hypotheses, whereby falsification of these hypotheses would result in the development of new knowledge, is an integral part of post-positivism.^11^ According to Mackenzie and Knipe,^11^ post-positivism aligns well with quantitative research methods (eg, experimental, and quasi-experimental). An individual who aligns with post-positivism can use the ICON framework to determine what important context attributes they need to measure before, during, and after implementation, and how these context attributes influence uptake of evidence or improvements in outcomes. Currently, we are developing an online repository of tools that measure attributes of context. The repository is intended to be used by both knowledge users and researchers and contains a decision support tool to aid individuals in choosing the tool that best fits their needs. We agree with Gagnon’s^5^ suggestion that the list of context attributes found in the study examining stakeholders’ tacit knowledge could be a basis for the development of: (1) a checklist or reporting guidelines for reporting contextual elements in implementation studies; or (2) a tool that measures the relative influence of specific context attributes in different contexts.

###  Social Constructivism Paradigm

 Social constructivism based on phenomenology and hermeneutics assumes that reality and knowledge are socially constructed, requiring understanding of individuals’ subjective interpretation of a phenomenon.^11^ Social constructivism assumes that research participants or the researchers interpret a phenomenon or information differently based on their identity or past experiences.^11^ Theories are often inductively generated through the interpretation of multiple subjective experiences.^11^ Individuals who align with social constructivism can use the ICON framework to determine, based on individuals’ experiences or roles, what they think are the most important or modifiable aspects of context in their setting, and at different phases of implementation. The ICON framework can be used to start conversations about context and relationships that may be particularly relevant to consider when implementing evidence-informed practice. According to Mackenzie and Knipe,^11^ social constructivism aligns well with qualitative research methods (eg, phenomenology and ethnography). We are working on a qualitative interview guide composed of open-ended questions based on the ICON framework that will help researchers and knowledge users begin discussions about context attributes and their prioritization.

###  Transformative Paradigm

 The transformative paradigm is based on criticisms that sociological and psychological theories, historically, did not address issues of social justice and marginalised people.^11^ According to Mackenzie and Knipe,^11^ individuals adopting a transformative paradigm align best with critical approaches to research (eg, critical theory or feminist theory). Individuals that align with a transformative paradigm can use the ICON framework as a starting point for examining context related to equity-deserving populations as well as in the Global South and primarily non-Anglophone countries. Furthermore, individuals can use the ICON framework to identify how current and historical power relations between individuals/groups, and how unequal and inequitable distribution of resources across settings and populations, may affect multiple context attributes across domains and levels of context. Considerations of equity, diversity, inclusion and intersectionality can be integrated in all the ICON attributes. The ICON framework outlines what attributes of context to consider when planning or implementing ways of meaningfully integrating these concepts in their research or during implementation. We agree with Cairney^2^ and Harvey^4^ who stated that access to resources plays an important role in implementation. We also believe that context could differ in non-English speaking countries as proposed by Eldh^13^ and Rycroft-Malone,^3^ and differ in countries considered as part of the Global South as proposed by Gagnon^5^ and van Pelt and Beidas.^10^

## Use of ICON Across Phases of Implementation

 We agree with several of the commentary authors^3,4,6,13^ who stated that context is not static; rather, it can change throughout phases of implementation and can be influenced by an implementation strategy or the introduction of a clinical practice. For this reason, we believe that the ICON framework context attributes should be evaluated before, during, and after implementation to identify changes in context over time.

## Conclusion

 The ICON framework was developed using a rigorous process aligning best with a pragmatist paradigm. The ICON framework advances conceptual clarity of the “context” concept in healthcare implementation as it provides the most comprehensive list of context attributes to date. The construction of such a comprehensive list of context attributes and their features is a necessary first step prior to conducting research examining interrelations amongst context attributes or research examining the moderating effects of context on implementation efforts. The list of context attributes is also a good starting point for individuals wanting to think about which context attributes and potential interactions between attributes are the most important to consider at specific phases of implementation. We posit that having a shared vocabulary describing the attributes of context can also facilitate mutual understanding and collaboration between researchers and knowledge users. The ICON framework can be used by individuals who align with post-positivist, social constructivist, and transformative paradigms and it can help individuals to be aware of the important context attributes at different phases of implementation.

## Ethical issues

 Not applicable.

## Competing interests

 Authors declare that they have no competing interests.

## Authors’ contributions

 JES, IDG, JB, JC, JJF, JMG, MH, NI, JL, SM, TM, and AMH conducted the study that examined system stakeholders’ tacit knowledge of context, which was the topic of the commentaries we respond to in this manuscript. JES, WJS, IDG, and AMH analyzed and interpreted the commentaries and conceptualized the structure of this manuscript. JES, WJS, IDG, and AMH drafted the manuscript. All the authors critically reviewed and revised the manuscript, approved the final version and agreed to be accountable for all aspects of the work.

## Funding

 The research studies we conducted to develop the ICON Framework were supported by two Canadian Institutes of Health Research (CIHR) Project Grants (Awards numbers: 326947 and 426122).
